# Consumer motivations and desired product attributes for 2.0 plant-based products: a conceptual model of consumer insight for market-oriented product development and marketing

**DOI:** 10.1007/s43546-022-00278-3

**Published:** 2022-08-01

**Authors:** Emma Beacom, Lana Repar, Joe Bogue

**Affiliations:** Present Address: Department of Food Business and Development, Cork University Business School, University College Cork, Cork, Republic of Ireland

**Keywords:** Plant-based product, Dairy alternative, Meat alternative, Product attributes, Product development, Market orientation

## Abstract

The plant-based product (PBP) market sector is rapidly growing, but there is a noted lack of qualitative data examining consumers’ perceptions of these products. This study aimed to examine consumers perceptions and usage of plant-based products and brands to further refine and extend our understanding of the different layers of contemporary plant-based consumption. Online consumer focus groups (*n* = 6) were used to gather qualitative data from consumers (*n* = 20) in Ireland and the United Kingdom. Qualitative analysis was conducted using NVivo v.26 where a content analysis procedure was used to reduce data into categories and sub-categories, after which data within categories was analysed to identify themes. Six overarching themes were identified: (1) Pro-social and moral motivations as most prominent in influencing PBP consumption; (2) Personal and sociocultural reasons as emerging motivators on PBP consumption; (3) Brand, ingredients, flavour and price as key attributes; (4) Natural, unprocessed PBPs as most appealing; (5) Replicating functional and nutritional properties of animal protein as more important than replicating sensory properties; and (6) Locally produced products and brands as preferred. A conceptual model of consumer insights required for market-oriented PBP development and marketing is produced. This model is consumer led, and confirms and extends/refines knowledge on motivations for consumption, evaluation of product attributes, and market gaps and improvements for a new generation of PBPs.

## Introduction

Plant-based products (PBPs), defined as food or drink products in which the main ingredient (or ingredients) is a substitute for meat or dairy products (Beacom et al. 2021), present significant competition and disruption to the global meat and dairy sector, with sales of PBPs in the United States increasing by 29% during 2018–2019 to $5bn, and the European meat substitute market forecast to reach €2.4bn by 2025 (Deloitte 2019; GFI 2020). Growth in the PBPs sector has been driven by the mainstream emergence of ‘flexitarians’, those who still consume meat and dairy but seek to reduce levels of consumption, and increased numbers of vegetarians and vegans. The increase in demand for PBPs has, therefore, in part, been fuelled by the trend for consumers to adopt these products as an occasional meal, drink or snack, or to make subtle swaps to their usual diet, rather than a complete change to a plant-based lifestyle (Mintel 2018). Multiple factors have been suggested to impact on this trend, such as a belief that PBPs can help achieve health goals; food safety scandals which increase consumer mistrust of meat products; increased diversity and availability of alternatives; and ethical and environmental reasons (Mintel 2018; Taufik et al. 2019; Ploll and Stern 2020). According to Mintel (2019a), one in five US consumers identifies as flexitarian; while in the United Kingdom, one in three (34%) meat eaters followed a flexitarian approach by reducing their meat consumption in 2018 (Mintel 2019b). In Ireland, research indicates that almost one-third (30%) of the population follows some kind of plant-based diet (i.e. adheres to a vegan, vegetarian, or flexitarian diet), and this is more prevalent among females, and younger age groups between the age of 18 and 44 (Bord Bia 2021). The PBPs market presents a significant opportunity for innovation for existing PBP producers and new market entrants.

There has been a noted increase in the number of ‘plant-based’ or ‘vegan/vegetarian’ products and ranges available on the marketplace, as food manufacturers and retailers respond to the increased demand for these products. Long-established brands, such as Quorn, have notably increased product line and depth, while several new brands and product lines of existing brands have been developed. For example, brands like Richmond and Birds Eye have increased their product line width to increase plant-based ranges, retailers such as Marks and Spencer and Tesco have added plant-based lines, and new market brands such as Beyond Burger and Strong Roots have been introduced on the marketplace.

Although vegetarian and vegan branded products are not new to the market, there has been an increasing shift in the nomenclature regarding how these products are described and marketed. The use of vegan claims on food is on the rise, with an almost 2% increase in the use of vegan claims on food between September 2013 and August 2018, while the use of vegetarian claims on food decreased (by approximately 2%) between 2013 and 2015, and has remained fairly stable since (Mintel 2018). There has also been a noted increase in the number of products and brands making reference to the ‘plant’ based origins of the food/beverage ingredients, with some popular brands and slogans including ‘Plant Power’ (Market-brand range of Tempeh based products), ‘Plant-Kitchen’ (Marks and Spencer own-brand range), and ‘Natural Energy Purely from Plants’ (Market-brand ‘Tenzing’ energy drink slogan).

In 2018, Mintel noted opportunity for private label to increase their vegan/vegetarian own-brand offering (Mintel 2018). Many retailers have since increased the diversity of their own-brand ranges by adding plant-based ranges, for example Marks and Spencer introduced ‘Plant Kitchen’ in January 2019 (Marks and Spencer 2019), Tesco introduced ‘Plant Chef’ in September 2019 (Chiorando 2020). These ranges have been well received by consumers, evidenced by strong sales (Weinbren 2019), and continuous retailer expansion of these lines (Engoli 2020; Askew 2021). Own-label ready meals are cited as a particularly prominent product category in contributing to the increased growth in the meat-free market, with own-label ready meal sales increasing by 45% between 2018 and 2019, valuing 38.3 million (Weinbren 2019). It appears that some retailers view plant-based foods as contributing towards their Corporate Social Responsibility goals in relation to increasing the availability of sustainable products, for example Tesco have pledged to increase PBP sales by 300% by 2025 to reduce the environmental impact of consumer shopping baskets (White 2020).

However, despite clear positive response to PBPs generally, there has been some controversy regarding the labelling and product descriptors used for these products. For example, Sainsburys introduced coconut-oil based cheeses in 2016, but faced backlash for using the term ‘cheese’ (Mintel 2018). A later ruling in the European Court of Justice in June 2017, banned the use of dairy terms like ‘cheese’, ‘milk’ and ‘butter’ for plant-based products (Curia 2017). It is noteworthy, therefore, that brands such as Oatly continue to describe their plant-based drinks as ‘milk’ in the US, e.g. ‘oat milk’; while in Europe, the same product is named as an ‘oat drink’ (Oatly 2020). There has also been controversy related to how meat alternatives are labelled, and a case related to meat-alternative naming was heard in the same European Court as that of the dairy term case; however in October 2020, the court conversely ruled in the favour of allowing labels such as ‘vegan burger’ and ‘vegan sausages’ (Abnett 2020).

Although some studies have been conducted to examine consumers opinions of PBPs, there has been limited research on the acceptability of these products among Irish and British consumers, and furthermore there has been limited qualitative study on this area. Qualitative insights are important to gain a deeper insight into consumer perceptions and behaviours. Research on market acceptance of foods often focuses on products and their specific attributes rather than considering patterns of product attributes which drive consumer acceptance (Palacios et al. 2009). This study, therefore, qualitatively examines consumer opinion on PBP product attributes through prompting discussion on a range of PBP consumption related elements (e.g. motivations, brand and product perceptions), rather than specifically prompting discussion solely on attributes. The overall aim of this study was to examine in depth consumers perceptions and usage of PBPs and brands, in order to revisit our understanding of the drivers of PBP consumption and ultimately inform more successful market-oriented PBP development.

## Theoretical underpinning

Rosenfeld and Burrows (2017a) constructed a ‘Unified Model of Vegetarian Identity’ (UMVI), presenting how plant-based food choice identity is influenced by externalised, internalised and contextual dimensions. The model purports that consumers have low control over contextual dimensions (such as situational factors like historical and timing conditions which influence on their plant-based dietary choices), and higher perceived control over internalised dimensions (such as motivations to consume them) and externalised dimensions (like dietary pattern). As consumers have limited control or engagement with contextual/situational factors (Rosenfeld and Burrows 2017a), the externalised and internalised dimensions are of more prominent interest from a food marketing perspective. The present study is in particular concerned with considering the internalised dimensions section of the model, primarily the ‘motivation’ dimension. The motivation dimension of Rosenfeld and Burrows (2017a) UMVI model was elaborated on in a subsequent paper by the authors (2017b), where they defined motivation as “*an internalization that informs one's goals, assigns personal meaning to one's food choices, and directly stimulates one to follow a dietary pattern*” (p. 460), and identified categories of motivations impacting on consumer PBP choice (pro-social, personal and moral). Nguyen et al. (2020) later developed a conceptual model related to plant-based food choice motivations (termed the SHOULD model), listing various factors which can motivate plant-based food consumption (Spirituality, Social relationships, Health concerns, Opulence of PB foods, Outlook on life, Understanding of human body structures, Love towards animals, Diet knowledge). The motivation dimension of the UMVI model is of interest as it is one in which consumers have control over, therefore consumers have a level of agency when shopping to choose certain products for certain reasons. Marketers’ knowledge of motivations (and barriers) can help to develop and market more consumer orientated products. Consumers will be motivated to choose PBPs for various reasons, and it is important for food producers to be able to understand the most important motives to address, as in order to develop a particular food market it is important to understand factors which impact consumers purchase behaviour in that sector (Peschel et al. 2019; Le-Anh and Nguyen-To 2020).

Further to internalised motivations, consumers can be influenced to consume (or not consume) PBPs by observing and making inferences about a range of extrinsic and intrinsic product attributes. Symmank (2019) and Hoffmann et al. (2020) reviewed the literature on the influence of key product characteristics which influence consumer decision making, summarising various key intrinsic and extrinsic attributes of importance. Extrinsic product attributes cited include country of origin, labelling, nutritional information, price, brand, convenience, packaging, reputation, and claims (Symmank 2019; Hoffmann et al. 2020), while intrinsic attributes cited include appearance, smell, taste and texture (Symmank 2019). Apostolodis and McLeay (2016) further identified product attributes of relevance for meat alternatives, the majority of which align with those identified by Symmank (2019) and Hoffman et al. (2020), and in addition suggesting ‘method of production’ (referring to organic or genetically modified food) as important attributes considered by consumers. DONE (‘Determinants Of Nutrition and Eating’) is a comprehensive interdisciplinary framework focused on exploring factors which drive consumption, integrating vast knowledge across many disciplines such as consumer research, psychology, public health and nutritional sciences (Stok et al. 2017). The DONE framework underpinned the work of Hoffman et al. (2020) and was also referred to in this study to allow for more breadth and depth when identifying desired product attributes and corroborating existing knowledge. Attributes identified by these authors and the DONE framework are used in the coding framework for this study, to examine the importance of these attributes to the consumers in our sample.

Although there has been much innovation in PBPs, there is a lack of knowledge about how best to promote these products to consumers (de Boer et al. 2017; Peschel et al. 2019). Identifying the motivations of consumers to purchase PBPs, as well as the product attributes they value, can help inform product development and marketing communications about the product, in order to increase likeliness of success on the marketplace (de Boer et al. 2017; Kempen et al. 2017; Peschel et al. 2019). Underpinning this is the theory of market orientation which premises that identifying and prioritising the needs of consumers and correspondingly developing their product or service around them will ultimately increase product sales and thereby profitability (Kohli and Jaworski 1990).

**Fig. 1:**
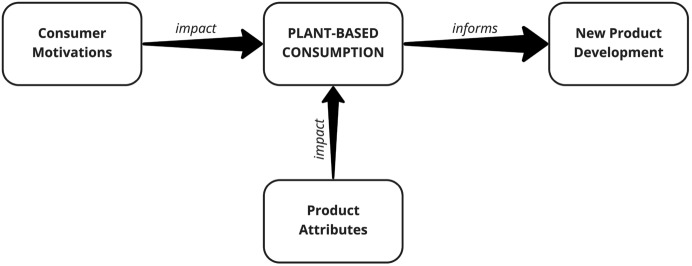
The study’s conceptual framework—a model of consumer insight required for market-oriented PBP development and marketing

This study premises that identifying and analysing data on consumer motivations, consumer perceptions of PBP attributes, and consumer opinions regarding market gaps and prospective product improvements, can inform future development of market-oriented PBPs, as conceptualised in Fig. 1. Findings from this study will be used to populate this conceptual model.

Therefore, the research objectives for this study are as follows: (1) to examine consumer perceptions of PBPs and their motivations for choosing these products; (2) to identify the importance consumer’s attach to various product attributes when choosing PBPs; and (3) to propose suggestions for development of market-oriented PBPs.

## Methods

Mini consumer focus groups (*n* = 6) were used to gather qualitative data from consumers (*n* = 20). Ethics approval for this study was granted by University College Cork Social Research Ethics Committee.

### Sample

Focus group participants (Table 1) were recruited during a previous study by the authors (a quantitative online consumer survey on PBPs (Beacom et al. 2021). At the close of the survey, participants were presented with an overview of the focus group aim and procedures and given the opportunity to indicate voluntarily if they would be willing to be involved. Those who indicated affirmatively that they were interested in participating in a focus group, and who provided their contact details for this purpose were contacted via email with a participant information sheet and consent form to consider further if they would like to be involved. Fifty-eight consumers who indicated they were interested in participating were contacted, and of these twenty opted to participate in the data collection. Consumers were assigned to focus groups as suited their availability. Due to participant availability, mini focus groups were conducted. Mini focus groups are a useful method of consumer insight when dealing with a specialised issue, as they provide more in-depth discussion than a larger focus group, while also being more efficient than individual interviews. They are also useful in a situation where the potential pool of participants is relatively small and difficult to reach, yet the research design requires that the phenomenon is discussed in an interactive group setting (Nyumba et al. 2018). As a convenience sampling method was used, representation among demographic characteristics is not equal; however, there was fairly good representation among age and gender (Table 1). Although it was intended that there would be greater representation from British consumers in the sample, participation from this group was more limited due to survey distribution (and related recruitment) constraints and participant availability. Most participants were regular PBP consumers and, therefore, possessed knowledge and experience highly relevant for consumer-led food innovation (Thomas et al. 2021).

**Table 1:** Demographic profile of focus group participants

Demographic characteristic	Total
*Age*	
18–25	4
26–39	7
40–54	7
55–74	2
*Gender*	
Male	7
Female	13
*Location*	
ROI	18
UK	2
*PBP consumption*	
Regular	16
Infrequent	4

### Data collection

Six mini focus groups, consisting of 2–6 participants per group were conducted online using Google Meet software. Focus groups were conducted over a four-week period in July 2020 and each lasted for approximately one hour. The group was facilitated by one researcher and a PowerPoint presentation used as an aid to display images and relevant text to encourage discussion. Groups were audio-recorded to facilitate transcription.

### Focus group guide

A semi-structured focus group guide was followed, consisting of six sections (for focus group guide overview and rationale, see Table 2). In *part one*, participants were asked questions relating to their perceptions of PBPs, and were then shown a definition of PBPs (Beacom et al. 2021) to contextualise the term for the purposes of this study. *Part two* examined consumers’ motivations for choosing these products. *Part three* assessed consumers’ PBP brand familiarity. Consumers were shown logos of market-brand and retailer own-brand (private label) PBPs, and asked questions relating to their familiarity with these brands, their perceptions and usage of these brands, and the availability of these brands in the stores they shop in. *Part four* involved product/brand comparisons. Consumers were shown two similar products from different brands across product categories (meat alternatives, dairy alternatives, ready meals, desserts and snacks), and asked to choose which they would be most likely to purchase, justifying their response with reference to their opinions on product attributes such as packaging, brand, appearance and price. *Part five* examined consumers’ perceptions of food claims on PBPs (e.g. health claims, sustainability/business responsibility claims, ‘free-from’ claims, and descriptors such as ‘plant-based’ and ‘vegan’). *Part six* involved discussion of PBP attributes, with the intention of co-creating new PBP concepts, or informing modification of current PBPs.

**Table 2:** Focus group guide topic overview and rationale

Section	Content	Rationale and relation to theoretical underpinning
1. PBP Introduction/Definition	Participant unprompted definition of what they think a PBP is Participant response to PBP definition for this study	To contextualise the study
2. PBP Choice Motivations	Participants asked their motivations for consuming PBPs	To investigate ‘motivation’ dimension in Rosenfeld and Burrows (2017a) UMVI conceptual model, and to investigate identified motivations in Nguyen et al.’s (2020) SHOULD model
3. PBP Brand Familiarity and Perceptions	Familiarity, usage, perceptions and availability of a range of PBP market brands and own brands	To investigate the attribute of ‘brand’ and other PBP attributes which emerged unprompted (Apostolidis and McLeay 2016Symmank 2019; Hoffmann et al. 2020)
4. PBP Brand/Product comparisons	Product choice comparisons	To investigate various PBP attributes which are important to consumers (Symmank 2019; Hoffmann et al. 2020; Apostolidis and McLeay 2016)
5. PBP Food Claims	Key food claims consumers look out for on PBPs Prompted discussion on PBP food claims of importance Prompted discussion of the naming of PBP products (i.e. plant-based versus vegan/vegetarian)	To investigate the attribute of ‘food claim’ and other PBP attributes prompted by the food claims discussed (e.g. nutritional content, sustainability credentials) (Symmank 2019; Hoffmann et al. 2020)
6. Co-creating a new PBP	Discussion on the importance of various product attributes (prompted with a list) when choosing PBP food and beverage products Identification of gaps in the PBP market/areas for improvement	To investigate various PBP attributes which are important to consumers (Symmank 2019; Hoffmann et al. 2020) To inform development of market-oriented products (Bogue et al. 2006)

### Data analysis

As the research objectives were primarily based on existing literature and a conceptual framework, a deductive approach to data analysis was most appropriate, to allow for findings to be analysed according to the predetermined objectives. A directed content analysis approach was determined to be the most appropriate method (Hsieh and Shannon 2005; Elo and Kyngäs 2008). A content analysis approach generally refers to an analytical method whereby codes emanating from the data are organised into categories aligned with the research objectives and conceptual framework, and thereafter the data are considered as a whole to identify themes (Erlingsson and Brysiewicz 2017). As a first step to analysis, data was transcribed ad verbatim and any identifying information removed. Prior to the focus group, participants were advised they could enter either their name or an assigned participant number when joining the meeting, this facilitated transcription as the researcher was able to differentiate between respondents when transcribing. Transcripts were then uploaded to qualitative data analysis software QSR NVivo (v.12). Transcripts were read and re-read to achieve data immersion, then coded using predetermined (*n* = 22) and emerging (*n* = 41) codes. A codebook was developed containing the predetermined codes (those based on the literature), and was added to as codes emerged from the data. Codes were analysed and deductively allocated into categories agreed upon by all authors (Motivations; Attributes; Product Development and Marketing) which aligned with the research objectives and the conceptual framework. A coding framework (Table 3) was developed to summarise and confirm all categories and codes which corresponded with existing literature and conceptual frameworks relating to the research objectives. Sub-categories were identified in accordance with the literature, and codes examined and deductively arranged into their relevant sub-category (Roller and Lavrakas 2015). Certain codes were discarded if they did not fit within the coding framework and were deemed not relevant, or alternatively were organised into a more appropriate code (e.g. codes relating to individual nutritional attributes such as ‘salt’ and ‘fat’ were combined into a ‘nutritional content’ node, in accordance with the literature). Codes within each category/sub-category were then checked to ensure alignment with their respective sub-category. Initial coding was conducted by one researcher and then checked by a second researcher. All decisions relating to the coding process and confirmation of the final coding framework and codebook were confirmed by all authors to increase validity and reliability of results. Codes within categories were inductively analysed to identify themes and provide context (Braun and Clarke 2006; Saldana 2008). Themes were refined, defined and named by all authors.

**Table 3:** Coding framework

Category	Sub-category	Codes	Source
Consumer motivations	Personal motivations^1,2^	Curiosity/Opulence^2^ Dietary pattern^1^ Allergy or intolerance* Feel good* Health^2^ Weight loss*	^1^Rosenfeld and Burrow (2017b) ^2^Nguyen et al. (2020) ^3^Symmank et al. (2017) *Own elaboration Although both Rosenfeld and Burrow (2017b) and Nguyen et al. (2020) discuss personal motivations, only Nguyen et al. (2020) explicitly named examples. Examples stated which align with codes have been indicated
	Pro-social and moral motivations^1,2^	Animal welfare^1,2^ Environmental concern^1^	
	Sociocultural motivations^2^	Household influence^3^ Recommendation from peers^3^ Cultural influence^3^	
Product attributes	Extrinsic^3,4,5,7^	Food claim^4^ Price^4,5,6^ Labelling^4,5^ Packaging^4,5^ Product information^4^ Nutritional information^5^ Origin of food^5,6^ Production method^6^ Brand^4,5,6^ Convenience^5^	^4^Symmank (2019) ^5^Hoffmann et al. (2020) ^6^Apostolidis and McLeay (2016)
	Intrinsic^3,4,7^	Appearance^4^ Texture^4^ Taste^4^ Ingredients^3,7^	^4^Symmank (2019)
Product development and marketing	Considerations for labelling*	Marketing ploy* Perceptions of PB terminology*	*Own elaboration
	Considerations for retailing*	Availability*	
	Considerations for product development*	Food preparation* Functionality* Innovation* New product idea* Protein substitute* Ready prepared products* Satiety* Sustainability* Trade-offs* Concerns PB not sufficient* Freshness*	

^*^Own elaboration = new codes developed from data. Rosenfeld and Burrow (2017b), Nguyen et al. (2020), and Apostolidis and McLeay (2016) = PB literature used to develop codes. Symmank (2019) and Hoffmann et al. (2020) = sources outside of the PB literature used to develop codes which corroborate current knowledge and ensure more comprehensive coding

## Results

**Fig. 2:**
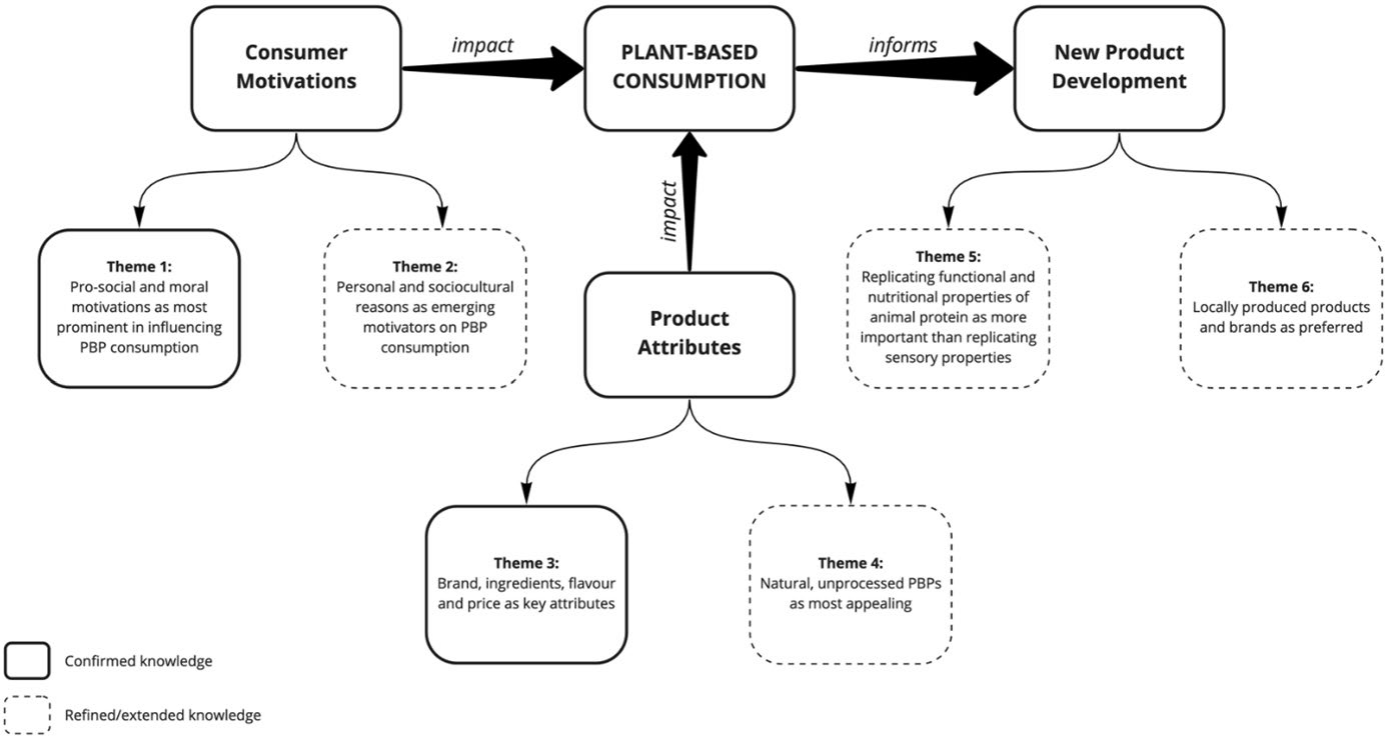
Plant-based 2.0—revisited knowledge on consumer insights for successful market-oriented PBP development and marketing

Codes within categories were examined, and six themes were identified, as summarised in Fig. 2, illustrating consumer insights relating to PBP consumption, which presents an overview of how consumer motivations and perceptions of PBP product attributes impact on consumption, and thereby inform new product development. Of those themes identified, two (Themes 1 and 3) confirmed existing knowledge, while four (Themes 2, 4, 5 and 6) refined/extended knowledge.

### Theme 1: pro-social and moral motivations as most prominent in influencing PBP consumption

Considering motivations as a whole, the most cited motivations for PBP consumption were pro-social and moral. Environmental concern was explicitly cited by seven participants as the reason they consume PBPs, while animal welfare concerns were cited by five participants.

*“It's all tied into kind of lessening the impact on the environment… and then it’s an animal welfare issue as well I think and a sustainability issue.*” (P8, 40–54, Female, Infrequent)

This environmental concern was evident not only when discussing motivations, but also in discussions related to product attributes (such as packaging and ingredients) and the products participants would like to see on the market:

*“[…] if I had a choice between two products and one had sustainable packaging and one didn’t, I’m more likely to choose the one with sustainable packaging.”* (P9, 26–39, Male, Regular)

“*If the product has palm oil, at least I try to see that it is from a sustainable source.*” (P6, 40–54, Male, Infrequent)

Further, when discussing brands, some participants (*n* = 6) indicated that they favoured or chose brands based on their environmental or socially responsible credentials:

*“…brand wise I would try and stick with the ones that were more sustainable. And …had responsibility over like their supply chain and everything like that.”* (P18, 18–24, Female, Regular)

Even when choosing PBPs, some participants discussed making sustainability related decisions regarding the types of PBPs they choose:

*“I try not to consume the almond milk or the soya milk, because they just have higher emissions like from an environmental point of view.”* (P18, 18–24, Female, Regular)

Some respondents (*n* = 3) further discussed how sometimes trade-offs had to be made when buying PBPs, for example buying a sustainable product, but it being in unsustainable packaging.

### Theme 2: personal and sociocultural reasons as emerging motivators on PBP consumption

Considering personal motivations, health, curiosity, and allergies/intolerance were important, with almost one-third (*n* = 6) of participants explicitly citing health as a motivation for consuming PBPs:

*“Mine is straight up health-based, I’ve just found as I’ve gotten older that meat doesn’t agree with me anymore. I have trouble eating it, digesting it, and when I do eat it I don’t feel too well afterwards, so yeah it would be pure health-based for me.”* (P13, 26–39, Male, Regular)

Five participants cited their curiosity about new PBPs as an incentive to purchase:

*“I am not vegetarian or vegan, so I normally consume these products based on some health aspects, and I really like to try different products. Every time that I go to a supermarket I want to buy at least one or two different things that I've never bought before.”* (P5, 40–54, Female, Infrequent)

Although sociocultural motivations (such as cultural upbringing, household influence, recommendations from peers) were cited, these were not as commonly discussed across the sample, as pro-social and moral motivations. Four participants cited their cultural environment or household influences as impacting on their consumption (or otherwise) of PBPs. Four participants mentioned being influenced to buy PBPs by peers, while one participant cited social media as influencing their PBP purchase behaviour.

### Theme 3: brand, ingredients, flavour and price as key attributes

The study participants quoted brand, ingredients, taste and price as important attributes of PBPs. The variety of products as part of a brand portfolio and the quality of those products was important to the participants:

“*They’re reliable brands [referring to specific PBP brands] I would always buy, because I know they are good products.*” (P1, 18–25, Female, Regular)

Some participants (*n* = 5) outlined that they regularly switched between brands as they recognised some brands had certain products developed better than others. Interestingly, five participants stated that they preferred a smaller, lesser known brand as opposed to a more developed, corporate-like brand:

*“The hero, you know [a bigger brand] like…it could be for the sake of mass production or high volume, they might be taking shortcuts in their processing or shortcuts here and there, and that's why I kind of [would] be a bit iffy about them to be honest, so I would be all for [a smaller brand].”* (P4, 18–25, Male, Infrequent)

When it came to ingredients, some participants highlighted that vegetables as core ingredients of PBPs are very attractive:

“*I’d love to see, I don’t know how practical this would be or how it could be done, a move away from soya and, you know, meat substitute products if they could use more natural veg rather than vegetable substitutes. (…) I’d love to see vegetarian sausages that might be based maybe on cauliflower or something more natural than soya.*” (P12, 40–54, Female, Regular)

The findings revealed that several respondents preferred to avoid soya for personal and/or environmental reasons (*n* = 3), stay away from palm oil (*n* = 2), and steer clear from sugar (*n* = 3) and highly salty PBPs (*n* = 2).

Flavour was considered as a critical factor for consumption of PBPs, but while some participants found it limiting: “*(…) particularly for me, its flavour is a key factor. I find a lot of these plant-based products don’t appeal to me at all in my flavour profile.*” (P6, 40–54, Male, Infrequent), others were willing to compromise: “*(…) I don’t mind sacrificing a little bit [of flavour] just for the benefits that I mentioned earlier [something that is sustainable]… that would be my ideal*.” (P4, 18–25, Male, Infrequent).

PBPs were perceived as more expensive and this was a concern for participants, as some of them opted for cheaper products (*n* = 8). Moreover, some participants raised an issue with keeping up with the price of PBPs due to their limited budget and a need for these products to become more affordable:

“*I think something else I would say is [the] cost [of] the [anonymised brand] stuff is ridiculously expensive, so we’re just buying whatever our budget allows, in terms of cost of meals for the week and that.*” (P9, 26–39, Male, Regular)

“*I think if they could make dairy alternatives in the line of cheese more affordable…I used to buy lovely cheese out of a health food shop, but it was nearly €4 for a small block of it, so I’m buying for a family so I couldn’t sustain that if I want to make sure I get my basics, like oat milk and things like that. So, if they could make things that are affordable, alternatives to dairy, the likes of cheese and stuff.*” (P12, 40–54, Female, Regular)

One participant suggested that PBP pricing, if perceived to be expensive, may be strategically linked to quality perceptions rather than directly related to the cost of ingredients, but also noted that they did not think this approach was necessary:

“*I used to work in the food industry so I kind of know that when you use vegetarian sources the product is cheaper and they just put up sometimes premium prices so people don’t think it’s too cheap [or] the quality [is not as good] but I think now as people get more confident about products they kind of have a changed mindset…so I’m very okay about lower prices because I know these products are mostly cheaper than meat products.*” (P5, 40–54, Female, Infrequent)

### Theme 4: natural, unprocessed PBPs as most appealing

Seven participants explicitly stated that they were interested in natural, unprocessed products: “*I don't want like meat replacements as much, as I do just kind of fresh food*” (P2, 26–39, Female, Regular), and some felt the processed, or unhealthy nature of some PBPs (being high in fat, salt or sugar) was a barrier to consumption:

*“I don't like it when I see, you know like, a really highly processed food and it says it’s plant-based, and I look at the ingredients and it's a lot of, you know, just not like whole products, it's just a lot of like substitutes in one. So it can be quite frustrating when I think I'm picking up something that might be healthier, but it's actually not.”* (P17, 18–24, Regular)

It was indicated that natural, healthy PBPs would be a favoured addition to the marketplace: *“I’m looking for something that’s healthy and like limited ingredients… I would love to have those just to grab and go and I just don’t think there’s enough right now like that* (P2, 26–39, Female, Regular)”. In particular, participants mentioned that they would like to see a greater selection, and greater availability, of meat substitutes that they could use to prepare meals from scratch:

*“The problem is that there are not many products and most of the time they are pre-prepared in some way, so instead of buying, um okay tofu you can find it, but there are many other products like seitan, it’s difficult to find it rogue and to use it and prepare something, you find it in something, whereas if you find meat you are you going to prepare it the way you like it and the same with plant-based products.”* (P14, 40–54, Male, Regular)

However, participants did acknowledge the convenience of ready prepared PBPs, P20: “*they're still quite handy to have you know if you've had a long day or something like, that*,”

“*Wouldn’t eat them every day but …sometimes it's just easier to have stuff like this in the freezer or the fridge*” (P2, 26–39, Female, Regular) and indicated that they may, in particular, appeal to those lacking cooking skills, or that pre-prepared PBPs removed the barrier to consumption of people being unfamiliar with how to cook these products or what to eat them with:

*“I think in the UK we’re really bad at cooking when making vegetables the centre of the meal...I think maybe in the UK we are looking for meat substitutes a bit more because we're looking to replace that meat and two veg kind of thing that gets drilled into us when we’re quite little.”* (P20, 40–54, Female, Regular)

### Theme 5: replicating functional and nutritional properties of animal protein as more important than replicating sensory properties

There appeared to be a desire for products to emulate meat or dairy in terms of functionality but not necessarily in taste, with some participants (*n* = 5) specifically stating that they would prefer that PBPs do not try to mimic the taste of meat, and instead focus on being innovative products of their own: “*I don't want to taste anything that tastes like meat.”* (P18, 18–24, Female, Regular).

*“Sometimes I find that there's too much of an effort to make the products very like meat, which maybe takes away from what could be very good vegetarian or vegan products in their own right.”* (P10, 40–54, Female, Regular)

They therefore considered that PBPs were more likely to be successful if they instead focused on “*[making] them into a more interesting product of their own*” (P10, 40–54, Female, Regular). Although consumers do not need alternatives to mimic the taste or texture of animal protein, mimicking functionality was indicated to be important for some product categories. PBPs lacking the functionality of their alternative was cited, or agreed upon, by almost one-third (*n* = 6) of participants as problematic. Functionality was discussed primarily in relation to dairy alternatives, for example it was felt by five participants that PB drinks did not froth or mix well in tea/coffee, and one participant noted the consistency and functionality of plant-based cheese not melting well “*consistency is quite different in a lot of them, they don't melt very well like dairy cheese*” (P20, 40–54, Female, Regular). It was indicated therefore that improvements to the functionality of certain dairy products would be a useful product development consideration: “*I’m always on the search for a plant-based beverage that goes well in tea*” (P2, 26–39, Female, Regular); “*I think there’s a lot of room for improvement of plant-based cheese products*” (P20, 40–54, Female, Regular).

In addition to considerations about how alternatives replicate functional properties related to preparation and consumption, there was some discussion about how the nutritional functionality of alternatives compared with that of animal protein, and some related concerns about the nutritional sufficiency of a plant-based diet:

*“I think one of the challenges that vegetarians have is trying to get that breadth of dietary requirements. So there are certain things that you get naturally from red meat and fish that it's hard to find with plant-based products or vegetables.”* (P9, 26–39, Male, Regular)

Protein in particular was discussed as a macronutrient which was important to participants (*n* = 4):

*“Well for me [an ideal product] would be add[ed] protein, the protein is one of the hardest things to get into you but it’s vital, especially if you do any type of training.”* (P13, 26–39, Male, Regular)

### Theme 6: locally produced products and brands as preferred

Although discussion indicated greater recognition of market brands, participants on the whole did not necessarily display any preference for branded PBPs and bought/were open to buying both own-brand and market-brand PBPs. Where preference was indicated for a particular market brand, it was generally related to brand familiarity, or price motivations, rather than because of negative perceptions about the alternative (own brand or alternative competitor market brand).

*“I usually try and go for what’s the cheapest, So I kind of don't, I don't usually think oh I need the [brand name] milk, I just need, I want soy milk… I'd probably just go for [brand name] because I'm more familiar with the brand and I think in UK where I live [brand name] is usually cheaper than [competitor brand name].”* (P17, 18–24, Female, Regular)

However, one participant associated lower priced own-brand products with lower quality nutritionally:

*“I mean I’m absolutely not biased in any way whatsoever between own brands and named brands, it’s just the own brand ones which would be more packed with salt because they’re trying to produce it cheaper, but if it was an own brand with the exact same ingredients…”* (P15, 26–39, Female, Regular)

Despite no clear stated preference for market brands versus own brands, participants did state a preference for locally produced products and brands, some linking this with the previously discussed theme related to sustainability:

*“If…the produce has been sourced from Ireland or whatever, I go for that before I go for something that has a lot of air miles and things like that on it and has had less time in the supply chain.”* (P18, 18–24, Female, Regular)

Almost half (*n* = 9) of participants discussed buying from local stores or preferring local produce, and five participants stated how their choice of retailer was influenced by whether or not they sold local produce and supported the local economy: “*I try not to shop in [stores] that aren’t bringing money back into the Irish economy*” (P18, 18–24, Female, Regular). Participants also indicated a willingness to pay more for local products:

*“I suppose when I see Fairtrade or a local business, I don’t mind spending[more]. Supporting local would be a big thing I’m interested in.”* (P1, 18–25, Female, Regular)

The paradox related to consumption of PBPs for sustainability reasons in terms of them being imported was mentioned by two participants:

*“…people tend to equate vegan or plant-based or whatever with being environmentally friendly, because that’s what’s kind of being pushed a lot, but it’s not going to be if you’re buying products from far flung countries and they’re using up huge carbon footprints to get here.”* (P6, 40–54, Male, Infrequent)

When considering market gaps, two participants suggested an Irish brand plant-based drink: “*It would be interesting if there was an Irish version of an oat milk being made*”. (P2, 26–39, Female, Regular).

## Discussion

### Consumer motivations, desired product attributes and new PBP development

This study examined consumer motivations and desired attributes of PBPs to refine and extend understanding of contemporary PBP consumption. The finding that pro-social and moral motivations (such as environmental or animal welfare concerns) were the most prominent in influencing PBPs consumption is also confirmed in the literature (Milfont et al. 2021; Wang and Scrimgeour 2021). This study also indicated that these motivations impact not only on the decision to consume PBPs but also influence decision making regarding specific products and brands (i.e. avoiding PBPs known to be less sustainable, and choosing brands perceived as being more pro-social and morally concerned). Considering the increased media and governmental communication regarding the importance of sustainable consumption and production behaviour, it is expected that the food choices of consumers will increasingly be motivated by concerns for the environment and its sustainability.

Further findings from this study, as well as more recent literature, indicate that consumers are increasingly choosing PBPs for a range of personal and sociocultural reasons. Although meat and dairy products have been traditionally considered as nutritious inclusions to a balanced diet, there has been a sociocultural shift towards promoting reduction of consumption of these products for health reasons, for example to lower the amount of saturated fat in the diet, or due to concerns about findings linking meat/fat consumption to higher incidence of certain cancers, stroke and heart disease (Richi et al. 2015). Consequently, health reasons are increasingly being reported as positively influencing the adherence to a plant-based diet (Fresán et al. 2020; Vizcaino et al. 2020). Maintaining a weight within recommended guidelines is an important aspect of health, and although weight control was not extensively discussed in this study, previous research supports weight control as a motivation for choosing PBPs (Dorard and Mathieu 2021). Therefore, it is suggested that pairing sustainability and wellness attributes of PBPs could attract more consumers, especially those looking for more natural and holistic ways to control weight. Further, the clear incentive of curiosity for trying PBPs, both in this study and in the literature (IFIC 2020; Estell et al. 2021; Beacom et al. 2021), reinforces the need for continued product development and innovation in this category. Continuous innovation is one of the key strategies for success on the plant-based market (Vlietstra 2021), however, affordability should go hand in hand with innovation, as PBPs were often perceived as expensive in the study and the participants frequently opted for a cheaper option, a finding supported by the literature (Apostolidis and McLeay 2016). It is difficult to accurately assess and make definitive comparisons between the affordability of PBPs and their meat or dairy alternatives, due to the complexity in comparing ‘like-for-like’ products and pricing across countries and brands, and it is acknowledged that often despite consumers perception that PBPs may be more expensive than animal protein alternatives, it is often the case where the reverse is true. Further, it may also be the case that consumers perceive PBPs (particularly meat alternatives) to be less affordable than meat products, when considering their comparative value and satiety properties, i.e. the comparative price of vegetables and other ingredients often used as a base for PBPs, and meat, and the comparative volume of meat alternatives versus meat that may need to be consumed for satiety.

This study confirmed known key intrinsic (ingredients and flavour) and extrinsic (brand and price) product attributes that were important to PBP consumers. Natural, unprocessed PBPs were preferred, with participants favouring vegetable ingredients at the core of PBPs and some avoiding soya, palm oil and PBPs high in salt and sugar for various reasons, such as taste, allergies, nutritional reasons, or sustainability concerns. Although soy-based alternatives were popular in the early stages of the plant-based phenomenon, the low preference for soya was confirmed in studies by Weinrich and Elshiewy (2019) and Rondoni et al. (2021). Accessibility to natural, unprocessed PBPs was, however, considered a problem by some, indicating the need for increased innovation and retail distribution of PBP options which can be used to prepare meals from scratch, rather than simply providing more ready meal options, which although acknowledged to be convenient, often do not accord with health conscious PBP consumers’ eating preferences.

Related to this, although consumers desired flavourful PBPs, they did not necessarily demand PBPs to taste like their meat/dairy alternatives. They preferred alternatives to be easily substitutable for meat/dairy products with regards to their functional and nutritional properties, i.e. they wanted alternatives that could similarly replace meat or dairy in a recipe or drink without compromising the quality of the eating experience, or that were unique products of their own, rather than unsatisfactory replicas of meat/dairy products. One of the biggest problems with PBPs is their lack of macro- and micro-nutrients which need to be added at the expense of higher costs (Scholz-Ahrens et al. 2020; McClements 2020). PBP manufacturers should, however, recognise that poor nutritional profile and usage functionality (e.g. product ability to melt or mix) in comparison to meat/dairy products can be potential barriers to consumption, and therefore should seek to overcome these in product formulations. While undoubtedly the trend of mimicking meat/dairy products will continue for some time, continued innovation in this category through novel and dynamic product ideas, packaging, convenience and branding could help PBPs to move away from being perceived as ‘alternatives’ or ‘analogues’, avoiding unfavourable comparisons between PBPs and meat/dairy alternatives. Moving away from labelling plant-based foods as only for vegetarians or vegans or defining them as part of a social identity (Plante et al. 2019), and instead creating a new category or fusions with categories like functional foods (Goyal et al. 2021), could yield higher acceptance by a wider audience and drive greater sales, especially among flexitarians and omnivores (Sloan 2021). Additionally, the utilisation of more market-oriented techniques, such as sensory and consumer acceptance analysis (Fiorentini et al. 2020) and AI-powered research, should drive the Plant-based 2.0 revolution to create new exciting products based on consumers' expectations and trade-offs regarding health, convenience, sustainability, and ethics. In parallel with the new knowledge about the brain-gut connection, an avenue worth exploring further is the postprandial effect of plant-based versus dairy and meat foods consumption, such as in Zhou et al.’s research (2021a; b), which could inspire the use of new ingredients in combination for better nutrient bioavailability and the design of compelling and unique marketing messaging for Plant-based 2.0.

Another direction for new PBPs is a recorded desire for locally produced products, as some participants were willing to pay a higher price for local PBP brands and some also recognised a paradox of sustainability when PBPs were highly processed and sourced from abroad with longer supply chains. The explanation might go back to previously identified consumers’ desire for local products related to a search for local identity, more traceability, ethical consumption, reduced carbon footprint and concern for animal welfare (Kasriel-Alexander 2014).

### Scanning the competition

While a plant-based diet might seem the optimal solution for today’s world, it is not the only option. From a market-oriented and NPD perspective, the closest current rival to the new generation of PBPs is organic foods, and the closest future rival to PBPs is fermented and cultured animal proteins (Kateman 2021). Although the sales of organic foods remain relatively small, they are increasing significantly in developed countries according to Desquilbet et al. (2018). Despite the obvious differences between PBPs and organic foods (i.e. PBPs do not require strict adherence to ‘organic’ credentials, and there are meat and dairy products labelled as organic), motivations to consume are similar. For example, organic food is perceived as healthier, environmentally friendly and more in sync with animal welfare (Van Doorn and Verhoef 2015; Bryła 2016; Hansmann et al. 2020; Dang et al. 2021). Nevertheless, scientists are still divided on whether organic products per se have superior qualities compared to conventional ones (Reganold and Wachter 2016; Mie et al. 2017; Popa et al. 2019). Just like with PBPs, the price, together with availability, seems to be the key barrier to more consumption of organic foods (Buder et al. 2014; Schipmann-Schwarze and Hamm 2020). The advantage that PBPs have over organic products is their less demanding and costly production, as well as their no-animals paradigm. Fermented and cultured animal proteins are novel products which are still undergoing research and development, and are not yet widely available on the market for consumption (cultured meat is currently only available in Singapore). Cultured meat, where animal cells are grown in a lab to produce meat, offers the benefits of meat (i.e. similar functionality, sensory aspects, and nutritional content), but offers a more sustainable method of production (Zhang et al. 2020). Fermentation, which does not use animal cells and instead uses microbes to artificially produce animal proteins, likewise provides benefits of animal protein, while also reducing sustainability and animal welfare concerns, and concerns about allergens (Karlund et al. 2020; Ismail et al. 2020). Fermented and cultured meat will particularly appeal to consumers who choose PBPs for reasons other than disliking the taste or texture of meat, and in the years to come are the product types most likely to present competition for the PBP category. However, it is likely that there will be barriers to widespread consumer adoption of these artificially manufactured animal proteins (e.g. food neophobia), and it is here that PBPs will have an advantage as being a more trustworthy and familiar option. The future success of PBPs will, however, depend on the ability of producers to innovate and further reduce the costs in this category to make it more accessible and desirable in comparison to other options.

In the coming years, as more consumers opt for frequent PBP consumption, there will likely be an urge to accommodate a variety of demands and create many diverse PBPs, putting pressure on food companies and their innovation capacities. Moreover, consumers have become better informed over the past decade, with more power to influence, serve as co-producers and add value for other consumers, and also more critical of production practices (Perkins and Fenech 2014; Dellaert 2019; Balcombe et al. 2021). Hence, the acceptance of new PBPs will more likely be successful where food producers adopt a market-oriented approach through integrating key consumer insights into the NPD process.

## Theoretical and practical implications, and limitations

This study used various frameworks to identify consumer motivations and desired product attributes for PBPs. Figure 1 illustrated the identified elements required for successful market-oriented new PBP development (consumer motivations and desired product attributes). However, the models (UMVI and SHOULD) developed specifically for plant-based foods mainly cover motivations. Even the newer models, such as MENF (‘Motivation to Eat New Foods’) (Nezlek et al. 2021), focus on motivations and omit product attributes. Hence, this study referred to additional PBP-specific and non-PBP-specific literature, including the DONE framework, to allow an exploration of more product attributes and identify codes to refine/extend knowledge on plant-based consumption. By combining several frameworks, the study corroborated existing knowledge and contributed to a better understanding of the PBP phenomenon. It is suggested that a specific Plant-based Choice and NPD Model should be developed, considering the elements in Fig. 1, which will assist in developing successful market-oriented PBPs.

The conceptual model produced from this study will assist in developing and marketing new PBPs. The findings are applicable for current, and prospective, PBP producers, processors, marketers, and retail buyers, who, as illustrated in the model, should be aware of confirmed and emerging motivations for consumption, and perceptions and preferences regarding product attributes, and use this knowledge to inform NPD and marketing. Specific ‘refined/extended’ knowledge gained from this study to practically inform PBP development includes the emerging motivators of personal and sociocultural reasons which can influence PBP consumption, these emerging motivations can impact both on product formulations (ingredients), but also on product packaging and product concepts, e.g. packaging options to fit varying needs, such as single serve and convenience options to suit single-person households and time-constrained consumers. The finding related to the emerging desire for natural and unprocessed PBPs, as well as the finding that consumers desire PBPs to replicate the functional and nutritional properties of animal protein, influences product formulations and also informs the need to emphasise these attributes where applicable when marketing PBPs in order to increase consumer acceptability. The finding regarding preference for locally produced products and brands influences brand creation, product/ingredient sourcing and brand partnerships. While many studies focus on developing new PBPs from a technological point of view (Sha and Xiong 2020; Flores and Piornos 2021; Schreuders et al. 2021), this study used a market-oriented approach by involving consumers and listening to their opinions on the category and desires for new products. This study presents a model which can be used as a guide for product development and further tested, for example using conjoint analysis and sensory analysis studies, to design products and marketing strategies which optimally appeal to consumers. Considering the growing global trend of PBP consumption, this conceptual model has relevance and applicability outside of Ireland and the United Kingdom, and testing in various locations could allow for comparisons and identification of similarities/differences in varying worldwide markets, informing product development, marketing and imports/exports of PBPs in various locations.

It is acknowledged that a limitation of this study is the relatively small convenience sample used, which was influenced by data collection constraints during the COVID-19 pandemic and a limited participant pool. However, the study nonetheless provides a valid contribution by presenting a model of consumer insights for successful market-oriented PBP development and marketing which can be tested and confirmed with larger samples and in different locations.

## Conclusions

Previous studies have identified key motivations and attributes driving plant-based consumption. Using a qualitative approach, this study revisited the accumulated knowledge to find out if any new, or refined elements, are emerging in this area to inform successful new PBPs for the next decade. The findings confirmed existing knowledge regarding motivations and attribute preferences, and identified emerging motivations and preferences relating to attributes, functionality, and origin of PBPs. This study therefore indicated the next stage of the plant-based phenomenon. Considering the emerging motivators of personal and sociocultural reasons on PBP consumption, the desire for natural, unprocessed PBPs, the desire for PBPs to replicate the functional and nutritional properties of animal proteins, and the preference for locally produced products and brands, various recommendations have been suggested for PBP development and marketing. In Plant-based 2.0, more attention should be given to motivations such as sustainability and health, functional and nutritional value, affordability, less processed, locally produced, convenient, but not bland products. The future lies in developing innovative, advanced and authentic plant-based products with the desired sensory properties and sustainability credentials for specific target markets. What will distinguish this new generation of PBPs from its dairy and meat competition, and give them an advantage on the market, is the overall health and sustainability appeal blended with innovativeness, convenience, affordability and strong branding and messaging.

## Data Availability

Data are not publicly available because informed consent was not obtained for data sharing. Data are available from the corresponding author upon reasonable request.
